# Material context moderates the occupation-adjusted association between Visualization and prefrontal hemodynamics during naturalistic design: an exploratory wearable fNIRS study

**DOI:** 10.3389/fnrgo.2026.1928775

**Published:** 2026-09-10

**Authors:** Yusuke Nagamori, Fumika Suzuki, Mika Isobe, Haruhi Miyoshi, Towa Shikata, Takuo Ando, Kazaru Yaegashi

**Affiliations:** 1 Tokyo City University, Yokohama, Japan; 2 Ritsumeikan University, Kyoto, Japan; 3 Tohoku University, Sendai, Japan

**Keywords:** design attitude, individual differences, naturalistic design, neuroergonomics, prefrontal cortex, proportional-time normalization, Visualization, wearable fNIRS

## Abstract

It remains unclear whether individual differences in a design-related disposition relate to prefrontal hemodynamics during naturalistic design, and whether the material context of the task moderates any such association. We addressed this using the Visualization subscale of the design attitude measure (DAM), a preference for externalizing ideas visually that we treat here as a continuous disposition beyond the designer job label. Thirty-nine working adults completed an open-ended block-building chair-design task using a constrained material set and a broader, higher-variety material set. Prefrontal total-hemoglobin change was recorded with a wearable two-channel fNIRS device (NeU HOT-2000; Fp1/Fp2; a single 810-nm wavelength; 10 Hz), and each self-paced trial was resampled onto a common proportional-time axis. Visualization was higher in designers than non-designers (*d* = 1.07). After adjusting for occupation, higher Visualization was associated with a greater broad–constrained Fp2 difference. This Condition × Visualization interaction was localized to a single window at 60–79% of the proportional timeline at Fp2 (cluster-corrected *p* = 0.013), with no cluster at Fp1; however, the formal between-channel comparison was not significant, so channel specificity was not established. Within this window, the effect remained significant in all 39 leave-one-out refits. The positive simple slope was present under the broad set but not the constrained set; the broad set was also rated as less difficult, more enjoyable, and more conducive to idea generation. Adjusting for occupation increased the whole-design estimate from β = +0.11 to +0.19, because occupation and Visualization related to the Fp2 difference in opposite directions (at a given Visualization level, designer status was associated with a smaller broad–constrained difference), a statistical suppression that separates the visual-externalization disposition from the occupational label; this whole-design estimate was sensitive to one participant. The findings indicate a material-context-dependent association between Visualization (a continuous disposition rather than a proxy for the designer category) and the prefrontal hemodynamic signal, and call for preregistered replication. De-identified data are openly available.

## 1 Introduction

Design attitude is a stable dispositional orientation toward open-ended, ill-defined problems (Boland and Collopy, 2004; Michlewski, 2008; Ando and Yaegashi, 2017). Ando et al. (2020) operationalized it as a 15-item self-report scale with a six-point response format and five dimensions (Experimentalism, Optimism, Visualization, Collaboration, Empathy; English items in Supplementary Material 1), hereafter the design attitude measure (DAM; Yaegashi et al., 2022). Of these five dimensions, only Visualization, the preference for externalizing ideas through visual representation, has been reported to distinguish design professionals from other white-collar groups (Ando et al., 2020). Design expertise centers on framing and solution-conjecturing strategies (Cross, 2004), and externalized visual representation is central to design cognition (Suwa and Tversky, 1997).

Designers score higher on average, but Visualization is a continuous disposition that also varies among non-designers rather than serving as a proxy for occupational category (Ando et al., 2020), and we treat it here as the focal individual-difference variable, examined after adjusting for occupation. Existing evidence for the scale, however, rests mainly on self-report and occupational comparison (Ando et al., 2020; Yaegashi et al., 2022); whether DAM dimensions relate to individual-level prefrontal hemodynamics during a design task, beyond occupational labels, remains untested. The available building materials shape the opportunities for externalizing visual ideas, so the association between Visualization and the accompanying prefrontal signal may become evident only in some material contexts. Because the task is self-paced, this association may also be concentrated in particular portions of the process rather than present throughout (Kato et al., 2018, 2021).

Neuroergonomics studies the brain and behavior in real-world, naturalistic, and everyday tasks, and wearable neuroimaging has extended this inquiry to the working brain outside the laboratory (Ayaz et al., 2013; Pinti et al., 2020). Cognitive neuroergonomics, in particular, investigates the neural bases of the cognitive processes engaged as people interact with real or synthetic systems, valuing ecological validity alongside experimental control (Gramann et al., 2021). Wearable functional near-infrared spectroscopy (fNIRS) tolerates movement and is suited to prefrontal measurement in occupational settings; it has been applied to occupational-workload assessment (Ferrari and Quaresima, 2012; Ayaz et al., 2013; Scholkmann et al., 2014; Pinti et al., 2020; Alyan et al., 2021; Gemmerich et al., 2025) and to prefrontal measurement during naturalistic, applied evaluation (Hirabayashi et al., 2024). Recent work continues this line, including fNIRS assessment of prefrontal and motor cortical activity during overhead work with competing cognitive demands (Tyagi et al., 2023) and a feasibility study of portable fNIRS for monitoring high-arousal emotional states among high-risk workers in safety-critical environments (Tian et al., 2026). Portable prefrontal fNIRS has been validated for cognitive-workload and working-memory tasks, with recent devices engineered for wearable, out-of-laboratory use (Siddiquee et al., 2020; Saikia et al., 2021). This portability and movement tolerance allow the prefrontal hemodynamic signal to be measured during a self-paced, open-ended design task rather than a simplified laboratory analog.

Creative generation is supported by distributed brain networks rather than a single region (Beaty et al., 2015), and prior studies of artistic and design generation have reported prefrontal involvement (Kowatari et al., 2009; Kato et al., 2018, 2021). This recruitment appears to vary with the context and form of idea generation. Kato et al. (2018) reported different prefrontal activation patterns between hand and computer drawing in a Finke pattern-generation task, with broader recruitment during computer operation, particularly in the right prefrontal cortex. Kato et al. (2021) further found greater prefrontal activation during the generation of novel design ideas than during the recall of typical forms. These findings indicate that prefrontal hemodynamics during design depend not only on whether a design task is performed, but also on how ideas are generated, transformed, and evaluated. This context dependence raises a further question at the individual-difference level: whether the association between a dispositional preference for visual externalization and prefrontal hemodynamics also depends on the material environment in which design unfolds. Design activity proceeds through problem–solution co-evolution and the continual reinterpretation of intermediate representations (Dorst and Cross, 2001). The available external visual stimuli can shape design problem-solving (Goldschmidt and Smolkov, 2006), so the generative and evaluative demands of the design process may shift as a design develops.

The two-channel forehead montage used here (Fp1, Fp2; 10–20 system) prioritizes wearability and movement tolerance. Because it records only two sites, we do not identify specific prefrontal regions anatomically but interpret the signal as a relative prefrontal hemodynamic index, following published reporting recommendations (Yücel et al., 2021). Based on prior evidence of greater right- than left-prefrontal involvement in artistic generation (Kowatari et al., 2009), together with reports of prefrontal involvement in design generation (Kato et al., 2018, 2021), Fp2 (right) was selected as the focal channel before analysis, with Fp1 (left) analyzed as the comparison channel. Rather than assuming channel specificity, we tested the difference between Fp1 and Fp2 directly. The material context also matters: the two material sets differ on several attributes at once, the broad set differing from the constrained, monochromatic set in piece count, color, and shape distribution.

We evaluated this question on an open-ended spatial design task, using DAM Visualization as the disposition of interest and testing whether it relates to the prefrontal hemodynamic signal at the individual level, beyond the designer job label. Prior fNIRS studies of design have shown that prefrontal recruitment varies across tasks, techniques, and modes of idea generation (Kato et al., 2018, 2021; Shealy et al., 2020). We extend this context dependence from differences between tasks to differences between individuals, asking whether the association between Visualization and prefrontal hemodynamics depends on the material set and varies across a self-paced design process.

Because the design task was self-paced, its duration varied widely across participants, so the recorded time series cannot be aligned on an absolute time axis. To examine when the association emerged, we combined proportional-time normalization with cluster-based permutation inference (Maris and Oostenveld, 2007), allowing process-level structure to be localized along an otherwise unsegmented, variable-duration design process.

Within this proportional-time framework, we addressed two questions:

Q1: Does DAM Visualization distinguish designers from non-designers?
Q2: Does the material set moderate the association between DAM Visualization and the prefrontal hemodynamic signal measured at Fp2, and when during the self-paced design process is this association most evident?

Occupation was used as the grouping variable in Q1 and as an adjustment variable in the Q2 regression models; Fp1 served as the comparison channel. Q2 was tested with a combined model treating the material set as a within-participant factor rather than comparing separate per-condition models (Gelman and Stern, 2006).

## 2 Methods

### 2.1 Participants

Forty working adults were enrolled: 20 design professionals (product, graphic, interior, or architectural design) and 20 non-designers (administration, sales, or non-design engineering). All were right-handed with normal or corrected-to-normal vision and no neurological history. One designer was excluded from the fNIRS analyses for task noncompliance; both trials ended prematurely (at 53 and 136 s) with concurrent persistent motion artifacts. This left 39 participants (19 designers and 20 non-designers). The groups differed in age (designers: M = 32.0 yr, SD = 4.6; non-designers: M = 28.9 yr, SD = 4.5; t(38) = 2.10, p = 0.042, d = 0.67) but not in sex distribution (designers: 14F/6M; non-designers: 8F/12M; Fisher's exact p = 0.111). Sex was self-reported and analyzed as a binary category; gender was not assessed. The task order was counterbalanced (10 per group per starting condition), and recruitment was via Tanseisha Co., Ltd. and the participating universities (Table 1).

**Table 1:** Participant characteristics by occupational group.

Variable	Designers (*n* = 20)	Non-designers (*n* = 20)	Comparison
Age, M (SD), yr	32.0 (4.6)	28.9 (4.5)	*t*(38) = 2.10, *p* = 0.042 ^*^, *d* = 0.67
Age range, yr	23–40	22–38	—
Sex (F/M)	14/6	8/12	Fisher's exact p = 0.111
Handedness	All right-handed	All right-handed	—
Task order (C-first/B-first)	10/10	10/10	Balanced

C, constrained monochromatic material set; B, broader, higher-variety material set. ^*^ p < 0.05.

The DAM was administered to all 40 participants; the fNIRS analyses used N = 39.

The study was approved by the Ethics Review Committee on Human Research of Tokyo City University (approval no. 2024-h08-02) and conducted in accordance with the Declaration of Helsinki. All participants provided written informed consent.

### 2.2 Block-building design task

**Figure 1:**
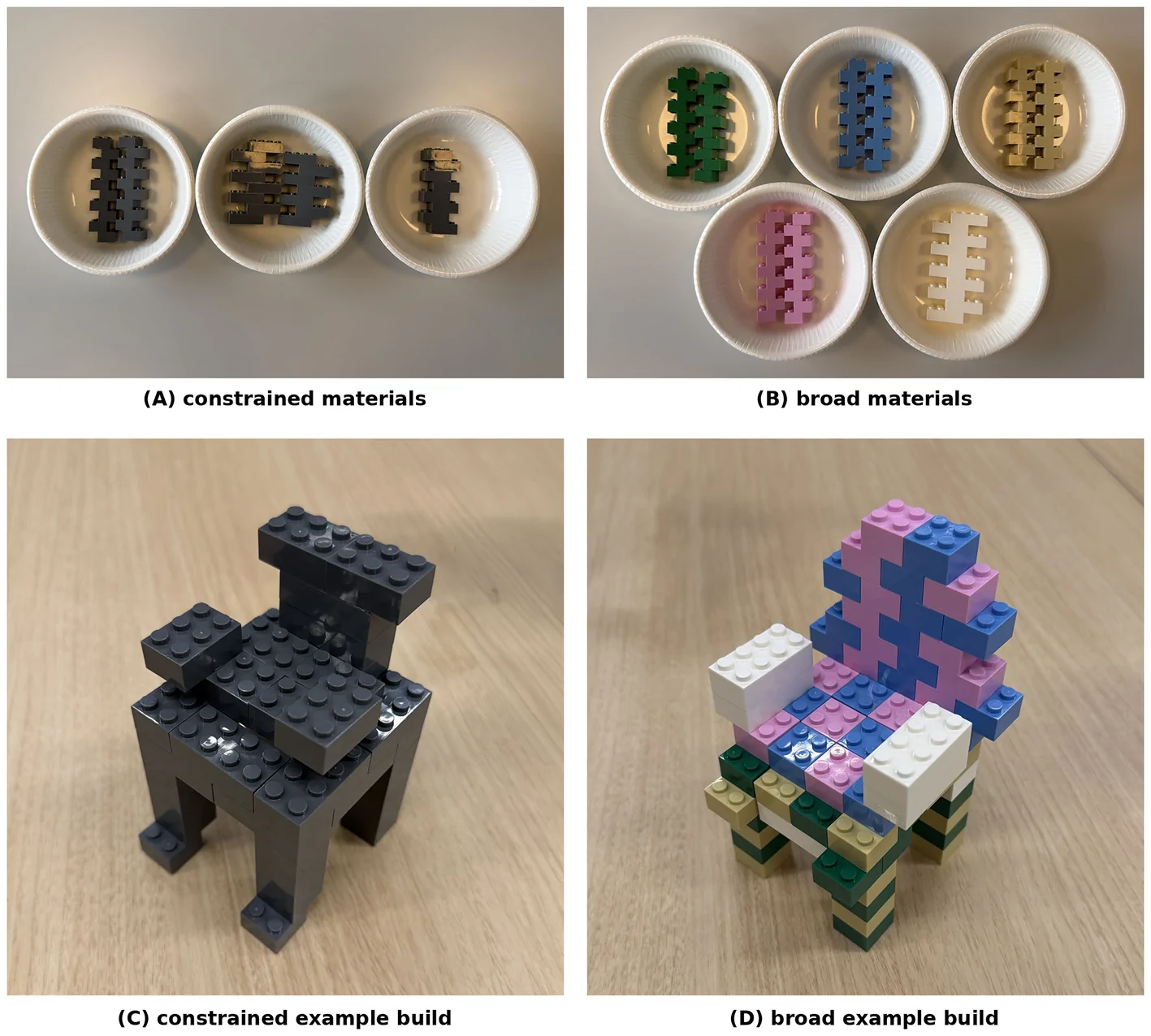
Task materials and example builds. **(A)** The constrained set (left, monochromatic cool-gray) and **(B)** the broad set (right, multicolor pastel) starter blocks, pre-sorted into separate bowls. Example completed chair builds for **(C)** the constrained set and **(D)** the broad set.

The block-building chair-design task (Nagamori et al., 2009) externalizes and revises ideas with physical blocks, making it accessible to participants without drawing skills. The constrained set uses a monochromatic, limited-piece kit; the broad set is a broader, higher-variety kit with more pieces and colors. Each participant completed two block-building chair-design trials in a counterbalanced order. The task was self-paced: participants were told to design “in about five minutes” but that this was not strict and that they need not attend to time; no clock was visible, and personal devices were removed for the session. Each trial ended when the participant declared completion. Trial durations therefore varied widely (constrained set: M = 414 s, SD = 219 s; broad set: M = 433 s, SD = 247 s; range ≈ 124–1137 s). Task onset and completion were marked online in the NIRS recording, and an approximately 2-min seated rest separated the two conditions. The constrained set used 45 monochromatic cool-gray interlocking blocks (20 of 2 × 2, 10 of 3 × 2, 15 of 4 × 2). The broad set used 90 multicolor pastel blocks: 80 of 2 × 2 in four colors (20 each of pink, light blue, green, and ivory) and ten white 4 × 2 units. The sets differed in color, piece count (45 vs. 90), and shape distribution (the 3 × 2 unit appeared only in the monochromatic set); these co-varying differences are considered in Section 4.4. Three design educators confirmed both sets allowed a wide range of chair constructions while remaining accessible without drawing skills. The working surface, lighting, and instructions were identical across conditions. Both sets and example builds are shown in Figure 1.

### 2.3 Wearable fNIRS measurement and signal processing

**Figure 2:**
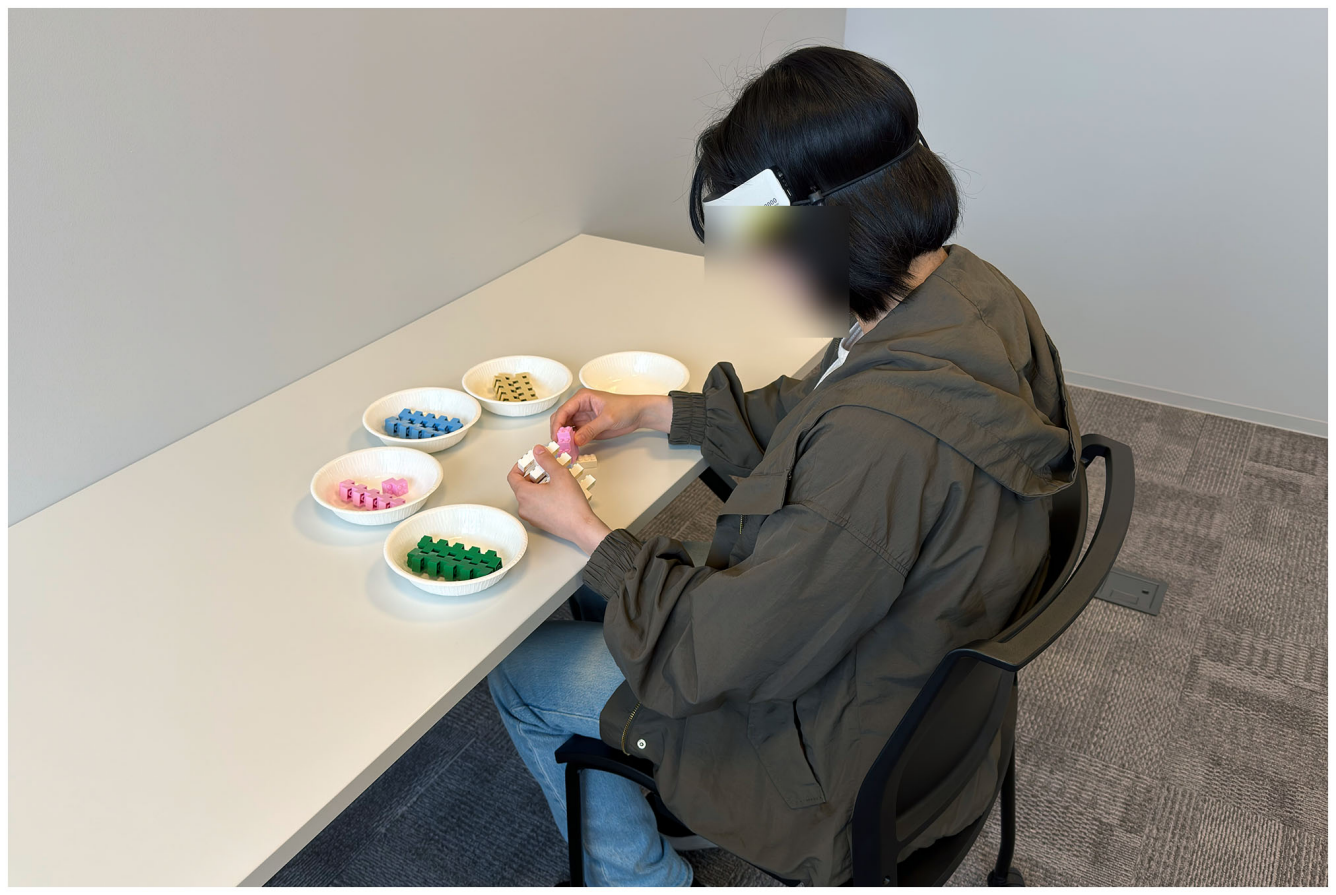
Experimental setup. A participant performs the block-building chair-design task while wearing the NeU HOT-2000 wearable fNIRS device.

Prefrontal hemodynamics were recorded with the NeU HOT-2000 wearable fNIRS device (NeU Inc., Tokyo; Figure 2) at Fp1 and Fp2 at 10 Hz using a single near-isosbestic wavelength (810 nm). Each sensor unit contained one LED and two photosensors. Samples flagged as artifactual by the device software (noise rate ≠ 0) were first linearly interpolated on the continuous signal rather than excluded (Supplementary Material 2). Flagged samples were rare (per-trial median 0.00%, maximum 12.14%); their proportion, duration, and distribution across participants and conditions, together with representative raw and corrected traces, are reported in Supplementary Material 2. Superficial contributions were attenuated by regressing the short source–detector signal (1 cm) out of the corresponding long source–detector signal (3 cm) at each site (static short-separation regression including an intercept; cf. Kiguchi and Funane, 2014), followed by linear detrending, yielding a short-separation-adjusted HbT signal; this step removed a median 74% of long-channel variance at Fp2 and 80% at Fp1, including most cardiac- and Mayer-wave-band power (Supplementary Material 2). Short-separation regression addresses superficial physiological contamination and is not a motion-correction method. The regression (adjusted long signal = long – [intercept + β × short], without a time lag) and the detrending were each estimated once per participant and channel over the continuous recording, before segmentation and baseline correction; the detrended residual was the HbT signal carried forward to baseline correction; no frequency filtering or smoothing was applied. The NeU HOT-2000 has itself been used as a prefrontal measure in peer-reviewed studies with a two-channel 810 nm forehead montage (Takahashi et al., 2022; Sasaki et al., 2026). Because a single near-isosbestic wavelength does not permit separation of oxy- and deoxyhemoglobin, the device yields only a relative total-hemoglobin signal (HbT). Cortical activation is typically accompanied by increases in both oxy- and total hemoglobin (Kamran et al., 2016), and HbT has also been used as a frontal hemodynamic measure in applied fNIRS research (Kim et al., 2022; see Supplementary Material 2). We treat this as background only: we make no claim that the residual signal specifically reflects cortical activity, and we interpret our findings throughout as associations with a prefrontal hemodynamic signal rather than as evidence of cortical activation. With superficial contributions attenuated, and because the measurement is continuous-wave under an assumed constant differential pathlength, this HbT signal was treated as a relative index of the prefrontal hemodynamic response in arbitrary units (a.u.) rather than absolute concentration (Delpy et al., 1988; Seiyama et al., 1988). Baseline correction subtracted the mean HbT during the immediately preceding 30-s eyes-closed rest, yielding the ΔHbT used in all analyses. Accelerometer- and gyroscope-based head movement and pulse rate were recorded concurrently and used only in sensitivity analyses.

### 2.4 Design attitude measure and subjective ratings

Design attitude was assessed using the 15-item DAM (Ando et al., 2020; Yaegashi et al., 2022) on a six-point scale: Experimentalism (four items), Optimism (three), Visualization (three), Collaboration (three), and Empathy (two); the English item content is reproduced in Supplementary Material 1. The internal consistency (N = 40) was: Experimentalism α = 0.66, Optimism α = 0.77, Visualization α = 0.72, Collaboration α = 0.68, Empathy Spearman–Brown ρ = 0.87; the focal Visualization subscale met the conventional 0.70 threshold. After each trial, participants rated perceived difficulty, enjoyment, ease of idea generation, and material sufficiency on a seven-point scale as a manipulation check. Throughout, Visualization denotes the DAM subscale score.

### 2.5 Statistical analysis

Occupational-group differences in the five DAM dimensions were tested with one-way ANOVAs (Holm-corrected across the five dimensions; Cohen's d). Post-trial subjective ratings were analyzed with linear mixed-effects models using by-participant random intercepts and Satterthwaite degrees of freedom, Holm-corrected across the four items. Because trial durations differed across participants, each trial was resampled by linear interpolation onto a common 100-point grid from onset to completion, so that all 39 participants contributed at every proportional-time point. This proportional-time normalization assumes that equal completion percentages index comparable positions in the self-paced design process; the limits of this assumption are considered in Sections 4.2 and 4.4, and duration-trimmed sensitivity analyses are reported in Supplementary Material 5. For the whole-design focal test, ΔHbT was averaged over each participant's proportional-time course within each condition, and the Condition × Visualization interaction was estimated by ordinary least squares (OLS) as the regression of the within-participant difference (ΔHbT under the broad set – ΔHbT under the constrained set) on grand-mean-centered Visualization and effect-coded occupation (Designer = +1, Non-designer = −1). The coefficient of Visualization is the focal interaction, tested against residual degrees of freedom (N – 3 = 36); this was the theory-driven focal test (Q2). Condition-specific (simple) Visualization slopes for the broad and constrained sets were obtained from separate per-condition regressions of the design-averaged ΔHbT on Visualization and effect-coded occupation. Both Fp1 and Fp2 were analyzed with the same model; the difference between Fp1 and Fp2 was additionally tested as a participant-level Channel × Condition × Visualization interaction, both on the design-averaged signal and time-resolved on the channel difference (Fp2 – Fp1) of the focal slope (Supplementary Material 3). For the time-resolved analysis, the same interaction was computed at each proportional-time point and tested across the timeline with a two-sided cluster-based permutation test (Maris and Oostenveld, 2007; Freedman–Lane residual permutation, family-wise-error-corrected); Fp1 and Fp2 were analyzed identically (Supplementary Material 4). Group ANOVAs and subjective-rating mixed models were computed in R using lme4 (Bates et al., 2015), lmerTest (Kuznetsova et al., 2017), and emmeans for estimated marginal means and simple slopes (Lenth and Piaskowski, 2026); effect sizes follow Cohen (1988). Proportional-time analyses (OLS and the permutation test) were computed in Python (NumPy/SciPy). We report effect estimates with confidence intervals alongside significance tests. Because ΔHbT is in arbitrary units, the regression coefficients are in signal units and are not comparable in magnitude across studies. We examined the focal model's residual normality and homoscedasticity, and the robustness of the interaction to heteroscedasticity-consistent standard errors and a case-resampling bootstrap; the interaction held under the bootstrap and the less conservative heteroscedasticity-consistent standard errors but attenuated under the more conservative small-sample estimators (Supplementary Material 4). The whole-design interaction was re-estimated with each participant omitted in turn, and the effect within the fixed 60–79% interval identified in the full sample was re-tested in the same leave-one-out refits (Supplementary Material 5). The time-resolved analysis used a distribution-free cluster-permutation test. Significance is marked throughout the tables and figures as ^*^ p < 0.05 and ^**^ p < 0.01; the group ANOVAs and subjective-rating comparisons are Holm-corrected, and n.s. denotes not significant. De-identified data and analysis outputs are openly available (Nagamori et al., 2026).

## 3 Results

### 3.1 Visualization is the only DAM dimension differentiating occupational groups (Q1)

**Figure 3:**
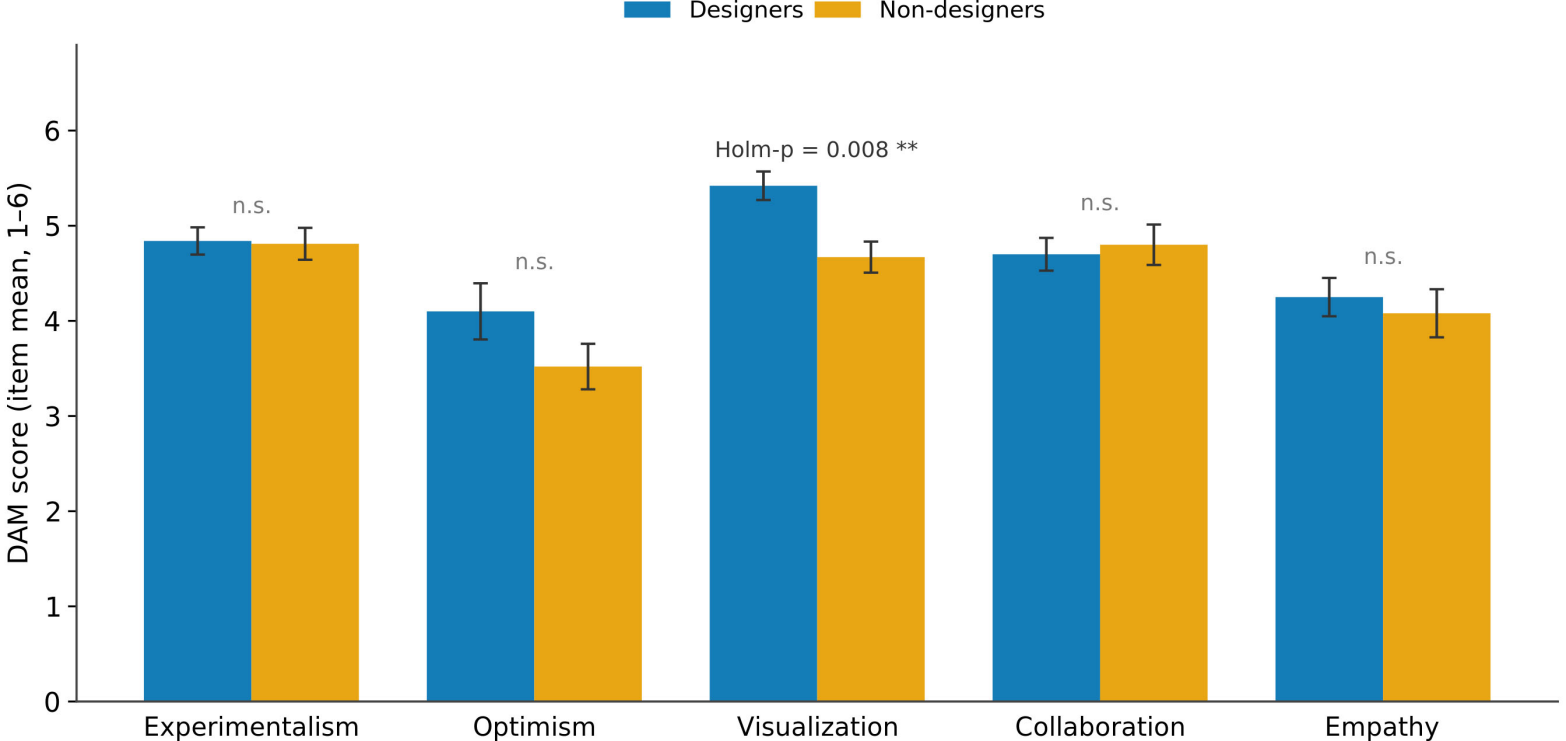
DAM scores by occupational group (*n* = 20 per group). Bars, group means; error bars, ± 1 SE. Significance markers reflect Holm-corrected *p*-values across the five dimensions (Table 2).

A one-way ANOVA with Holm correction identified an occupational difference on Visualization only (F(1, 38) = 11.47, raw p = 0.002, Holm-p = 0.008): designers: M = 5.42 (SD = 0.67) versus non-designers: M = 4.67 (SD = 0.73); mean difference 0.75, 95% CI [0.30, 1.20]; d = 1.07; partial η^2^ = 0.23. The other four dimensions did not differ after correction (all Holm-p > 0.53; Table 2, Figure 3). Visualization had been designated a priori as the focal disposition variable; the Q1 result is therefore consistent with, rather than the basis for, that choice. The design group was significantly older, by about three years (Table 1), but adjusting for age and sex did not change the Q1 or Q2 results (Supplementary Material 5).

**Table 2:** DAM scores by occupational group.

Dimension	Reliability	Designers M (SD)	Non-designers M (SD)	F(1, 38)	Holm-p	d
Experimentalism	α = 0.66	4.84 (0.64)	4.81 (0.75)	0.01	0.910	0.04
Optimism	α = 0.77	4.10 (1.32)	3.52 (1.07)	2.35	0.534	0.48
Visualization	α = 0.72	5.42 (0.67)	4.67 (0.73)	11.47	0.008 ^**^	1.07
Collaboration	α = 0.68	4.70 (0.77)	4.80 (0.95)	0.13	> 0.999	−0.12
Empathy	ρ = 0.87	4.25 (0.90)	4.08 (1.13)	0.30	> 0.999	0.17

Cohen's d, Designers–Non-designers. ^**^ p < 0.01.

### 3.2 Subjective task evaluation: material-set differences in task experience

**Figure 4:**
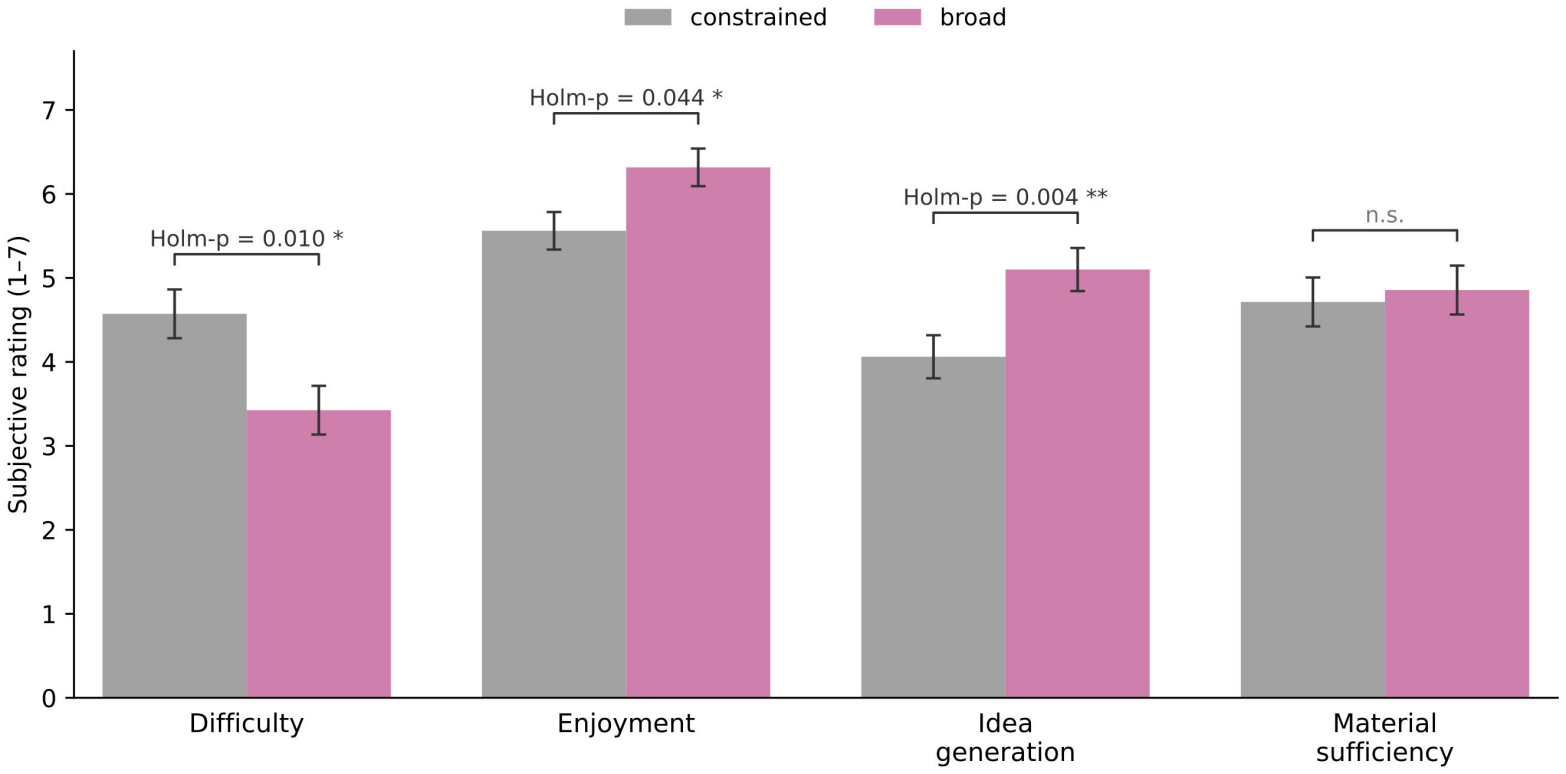
Subjective task ratings by material set (model-based mean ± 1 SE; 7-point scale). Brackets show Holm-corrected p-values across the four items; occupation showed neither a main effect nor an interaction with condition (all *p* > 0.18).

Compared with the constrained set, the broad set was rated as less difficult (Holm-p = 0.010), more enjoyable (Holm-p = 0.044), and more conducive to idea generation (Holm-p = 0.004); perceived material sufficiency did not differ (Holm-p = 0.610; Figure 4). No occupational main effect or Occupation × Condition interaction reached significance for any rating (all p > 0.18). Thus, participants reported different task experiences across the two material sets.

### 3.3 The material set moderates the association between Visualization and Fp2 hemodynamics (Q2)

**Figure 5:**
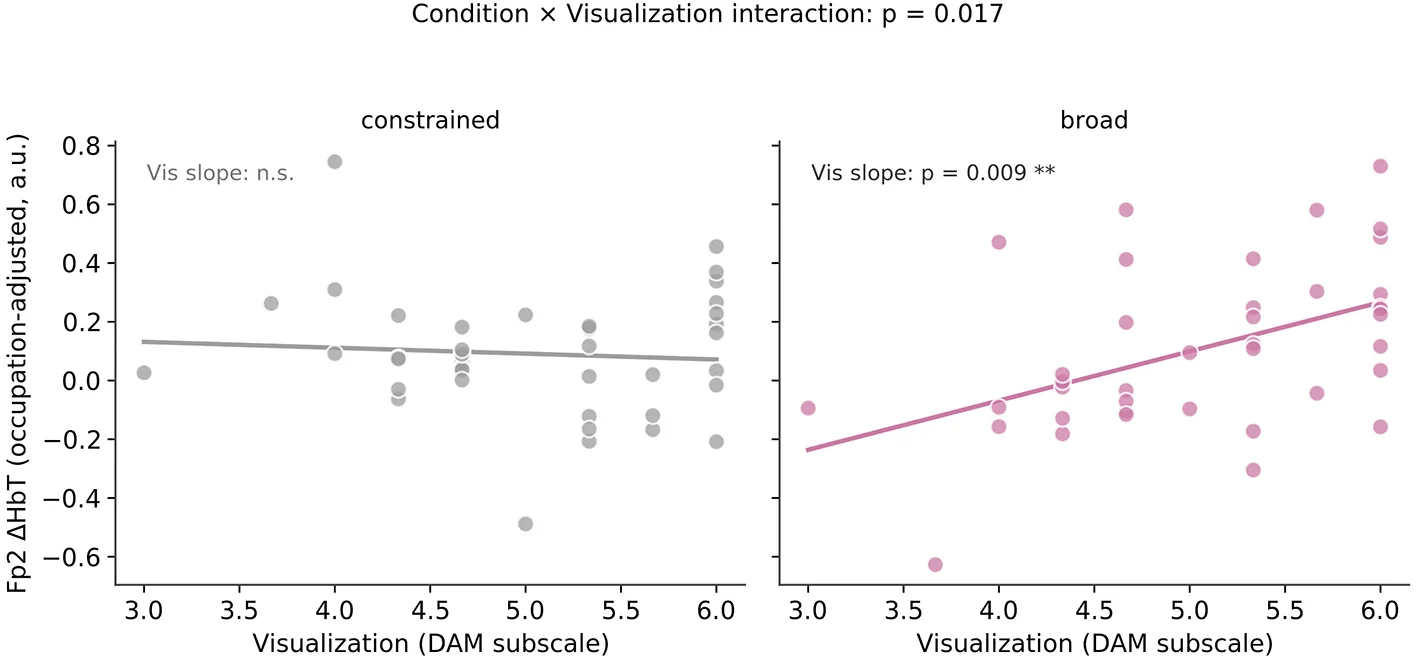
Visualization moderation of the Fp2 signal. Each point is the occupation-adjusted partial residual of design-averaged Fp2 ΔHbT (proportional signal) for one participant, plotted against the Visualization subscale score for each material set (the constrained set, gray; the broad set, pink). Lines are per-condition regression fits. The Condition × Visualization interaction was positive (*p* = 0.017); the broad set simple slope was positive (*p* = 0.009), whereas the constrained set slope was not (n.s.).

After adjustment for occupation, the association between Visualization and the Fp2 ΔHbT signal (a relative, single-wavelength hemodynamic index; Section 2.3) differed between material sets (Figure 5; Table 3). On the design-averaged proportional signal, the occupation-adjusted Condition × Visualization interaction at Fp2 was positive (β = +0.19, 95% CI [+0.04, +0.34]; p = 0.017): the broad set simple slope was positive (+0.17, p = 0.009), whereas the constrained set slope was not statistically significant (p = 0.706). At Fp1, the corresponding estimate was not statistically significant (p = 0.614). In a model containing occupation but not Visualization, the Occupation × Condition interaction was not significant (p = 0.396). In the joint model, however, the partial occupation coefficient was negative and statistically significant (β = −0.12, p = 0.044), opposite in sign to the Visualization coefficient. This pattern indicates statistical suppression arising from the correlation between Visualization and occupation (Supplementary Materials 5 and 6): the unadjusted (occupation-free) interaction was smaller and not significant (β = +0.11, 95% CI [−0.03, +0.24], p = 0.116), so the occupation-adjusted estimate is a partial association. The statistical significance of this whole-design estimate was sensitive to the deletion of a single influential participant: across the leave-one-out analyses, the interaction remained positive in all 39 refits but reached p < 0.05 in only 38 of the 39 refits (Supplementary Material 5). This contrasts with the time-resolved result (Section 3.4). The whole-design interaction held under a case-resampling bootstrap but not under the most conservative small-sample standard errors (HC2/HC3); these sensitivity analyses are summarized in Table 3 and Supplementary Material 4. We then examined when this association was most evident across the design.

**Table 3:** Material-set moderation of the association between Visualization and Fp2: estimates, temporal localization, and robustness.

Effect (Fp2, occupation-adjusted)	β	[95% CI]	*p*
Condition × Visualization, whole design	+0.19	[+0.04, +0.34]	0.017 ^*^
Simple slope, broad set	+0.17	[+0.04, +0.29]	0.009 ^**^
Simple slope, constrained set	−0.02	[−0.13, +0.09]	0.706
Fp1 (left), Condition × Visualization	+0.04	[−0.13, +0.22]	0.614
Temporal cluster, ≈ 60–79% (two-sided FL)	—	—	0.013 ^*^
Robustness— whole-design interaction			
Unadjusted (occupation-free)	+0.11	[−0.03, +0.24]	0.116
Case-resampling bootstrap (5,000)	+0.19	[+0.02, +0.35]	—
HC-consistent SE (HC0–HC3)	—	—	0.033/0.040/0.055/0.085
Leave-one-out (whole design)	Positive in 39/39 refits	—	Significant in 38/39 refits
Leave-one-out, fixed 60–79% interval	—	—	Significant in 39/39 refits

**Figure 6:**
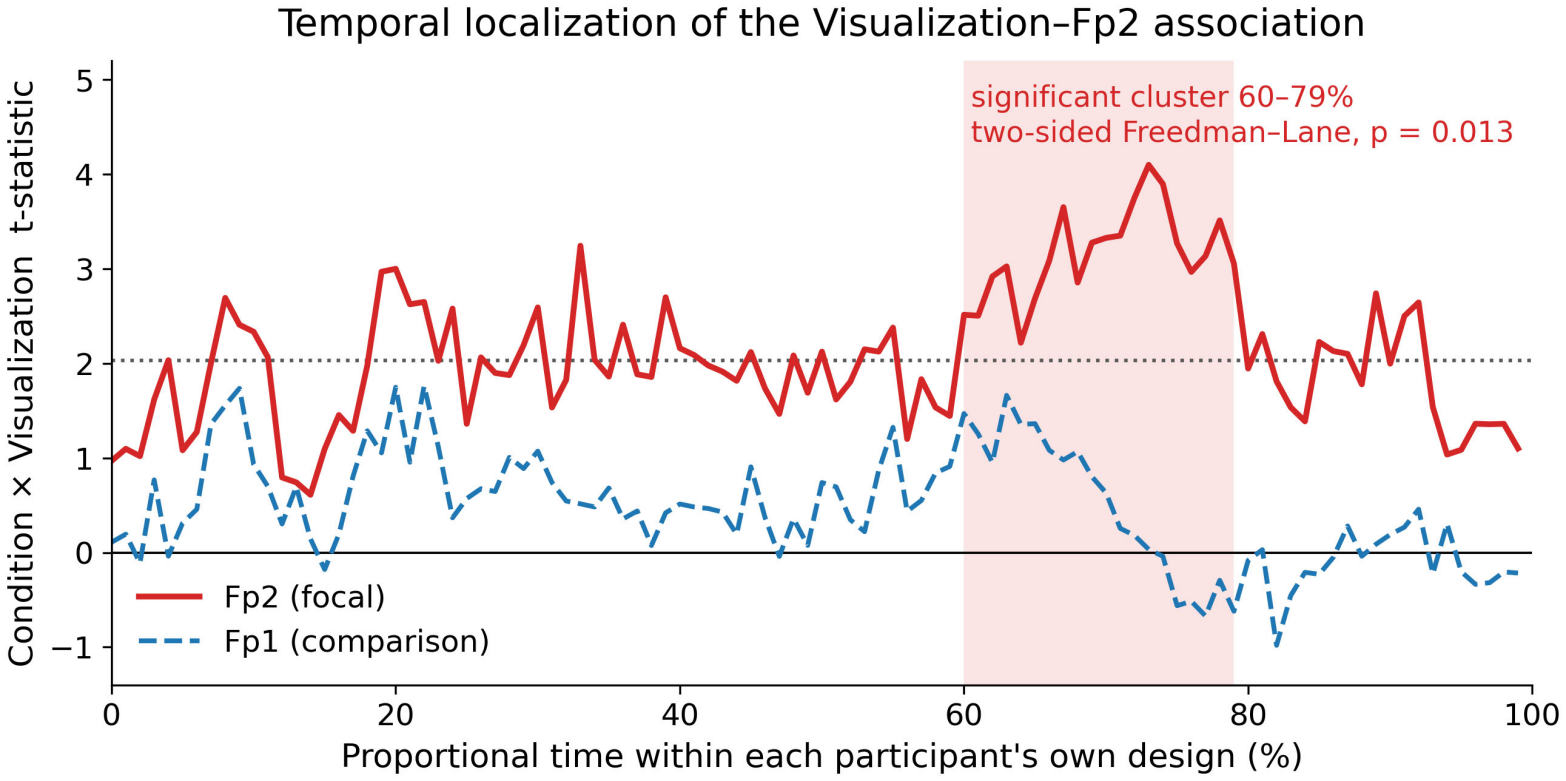
Temporal localization of the association between Visualization and Fp2. The plotted traces are the time-resolved t-statistics of the occupation-adjusted Condition × Visualization interaction (not ΔHbT time courses) across within-participant proportional time (0–100%), for Fp2 (right, red) and Fp1 (left, blue). The dotted line marks the cluster-forming threshold; Fp2 is solid and Fp1 dashed. The shaded band marks the single significant cluster (≈60–79%; two-sided Freedman–Lane cluster permutation, *p* = 0.013); no cluster was identified at Fp1. The formal time-resolved difference between Fp2 and Fp1 did not reach statistical significance (*p* = 0.056).

Occupation-adjusted estimates from the design-averaged proportional signal (*N* = 39, ordinary least squares, df = 36); the lower block summarizes robustness of the whole-design interaction (case-resampling bootstrap, heteroscedasticity-consistent standard errors HC0–HC3, and leave-one-out refits; Supplementary Materials 4–5). 95% confidence intervals are shown for all regression coefficients. The temporal cluster is the single significant cluster on the 0–100% proportional-time axis (two-sided Freedman–Lane permutation; Figure 6). In a model containing occupation but not Visualization, the Condition × Occupation interaction was not significant (*p* = 0.396); in the joint model, the occupation coefficient was negative and significant, opposite in sign to Visualization (*p* = 0.044; Supplementary Material 6). ^*^ p < 0.05, ^**^ p < 0.01.

### 3.4 The positive association is temporally localized to the mid-to-late portion of the task (Q2)

The tendency for higher-Visualization participants to show a larger Fp2 signal difference between the broad and constrained sets varied over the course of the task. On the proportional-time axis, the Condition × Visualization slope at Fp2 was predominantly positive across the proportional timeline; a two-sided cluster-based permutation test (Freedman–Lane residual permutation preserving the Visualization–occupation structure) identified a single significant cluster spanning approximately 60–79% of the timeline (cluster mass 63.2, max|t| = 4.11 at 73%, cluster-corrected p = 0.013), with no corresponding cluster at Fp1 (Figure 6). At the design-averaged level, higher Visualization was associated with a greater Fp2 signal under the broad set but not under the constrained set (simple slopes, Table 3). A formal participant-level Channel × Condition × Visualization interaction was not significant, either over the whole design (β = +0.14, p = 0.112) or in the time-resolved test (channel difference Fp2 – Fp1, two-sided p = 0.056; Supplementary Material 3). An association was thus observed at Fp2, while reliable channel or hemispheric specificity was not established. The condition difference itself, averaged over participants, showed no significant temporal structure independent of Visualization, indicating that the temporal effect was carried by the Visualization interaction rather than by a uniform task-evoked time course (Supplementary Materials 5 and 6). As an influence check, the estimate within the sample-defined 60–79% interval remained significant after omitting each participant in turn (the interval from the full sample was held fixed; all two-sided permutation p < 0.05) and was preserved under zero-phase low-pass filtering (Supplementary Material 5). Both the whole-design interaction and the fixed-interval effect were also robust to excluding participants with the shortest and longest trial durations under three trimming rules (whole-design β = +0.191 to +0.225, p = 0.024–0.027; fixed-window Freedman–Lane permutation p ≤ 0.004; Supplementary Material 5). The interaction also remained significant after controlling for concurrently recorded head movement, pulse rate, and trial duration, and Visualization did not predict the between-condition difference in any of these measures (Supplementary Material 5).

## 4 Discussion

### 4.1 Main finding and interpretation

Under the broad material set, higher Visualization was associated with a greater Fp2 ΔHbT signal during naturalistic design, whereas under the constrained set no such association was detectable; higher Visualization thus corresponded to a larger broad–constrained Fp2 difference. In the joint model, the partial occupation coefficient (occupation conditional on Visualization) was associated with a smaller broad–constrained difference. Visualization and occupation were positively correlated, but their partial associations with the broad–constrained Fp2 difference were in opposite directions. Adjusting for occupation therefore increased the Visualization coefficient from +0.11 to +0.19, a statistical suppression pattern (Tzelgov and Henik, 1991). Because both variables entered the same regression, the occupation-adjusted Visualization slope need not match a simple designer–non-designer contrast. This model therefore concerns sensitivity to the material context rather than the overall level of the Fp2 signal, and does not identify the underlying mechanism.

Read this way, the suppression is of substantive interest rather than a nuisance. Visualization and the occupational label were positively correlated, yet they related to the material-context sensitivity of the Fp2 signal in opposite directions: at a given level of Visualization, being a designer was associated with a smaller broad–constrained difference, whereas the disposition itself was associated with a larger one. The dispositional preference therefore carried information about the prefrontal signal that the occupational label did not, and that the label partly masked. This dissociation is consistent with the premise of the study, that Visualization is a continuous disposition rather than a proxy for the designer category, and it suggests that DAM Visualization scores were associated with context-dependent variation in the prefrontal hemodynamic signal over and above the occupational label. We offer this as an interpretation to be tested rather than an established mechanism: the estimate rests on conditioning two correlated predictors in a modest sample, and a design that samples the full range of Visualization within each occupational group would test it directly.

Fp2 was prespecified as the focal channel because prior work reports greater right- than left-prefrontal involvement in artistic generation (Kowatari et al., 2009). Earlier fNIRS studies compared the mean level of task-evoked activation (oxy- and deoxy-hemoglobin) as prefrontal engagement changed with the mode and demands of idea generation (Kato et al., 2018, 2021); the present study instead examines a relative total-hemoglobin signal and asks whether its association with an individual disposition changes with material context (a Condition × Visualization interaction rather than a group-level mean), and so complements rather than replicates that work. The direction is broadly compatible with prior work implicating prefrontal involvement in design generation, including right-prefrontal findings (Pidgeon et al., 2016; Shealy et al., 2020; Guo et al., 2023). However, neither Fp1 nor a formal Channel × Condition × Visualization interaction reached significance, over the whole design or in the time-resolved test (Supplementary Material 3), so this convergence is only suggestive and does not support reliable right lateralization.

The whole-design analysis was the theory-driven focal test, but its estimate requires cautious interpretation: statistical significance was sensitive to one participant and to conservative small-sample standard errors, whereas the effect within the fixed 60–79% interval held across all leave-one-out refits. DAM Visualization, which distinguished designers from non-designers in Q1, provides a brief, low-cost measure of a design-related disposition across both groups, so the present Q2 result provides preliminary evidence of a process-level association, adding to prior known-groups findings. The low-burden wearable two-channel device allowed prefrontal recording throughout a freely paced, hands-on design task ill-suited to fMRI, showing both whether and when the association occurred. Future field applications may therefore need to account for occupation or a comparable background variable. This individual-differences perspective also connects with work beyond the DAM: the object-spatial-verbal model distinguishes stable individual styles in object imagery, spatial imagery, and verbal processing (Blazhenkova and Kozhevnikov, 2009), field cognitive style and scene layout interacted on middle-frontal responses during a spatial updating task (Li et al., 2026), wearable fNIRS can capture individual differences in prefrontal activity (Nozawa and Miyake, 2020), and a professional design background was associated with distinct frontal and parietal activation patterns during human-AI co-creation (Wang et al., 2026). Relating DAM Visualization to such general style and background measures is a concrete direction for the planned confirmatory work.

### 4.2 Temporal localization and material context

The positive Visualization–Fp2 association was localized to approximately 60–79% of the proportional timeline (mid-to-late). Because the task was continuous, with no formal stages or behavioral phase markers, this is a location on the participant's normalized task trajectory rather than a defined design stage or a common physiological state. The localization is conceptually compatible with Kato et al. (2021), who compared prefrontal activation between generating novel design ideas and recalling typical forms; the present task, however, included no decision marker, and the measured sites were not anatomically equivalent. Unlike earlier work that located temporal effects across experimenter-defined stages or successive ideas, here the structure was recovered on a continuous, unsegmented self-paced timeline, and it was the individual-difference association, not mean activation, that localized. One speculative possibility, which cannot be tested with the present data because no behavioral markers of ideation, evaluation, or decision were recorded, is that the interval corresponds to a shift from idea generation toward evaluation or refinement: later ideas tend to be more original (the serial-order effect; Beaty and Silvia, 2012), and evaluative thought recruits networks distinct from those supporting generation (Ellamil et al., 2012). We retain this only as a hypothesis for confirmatory work with behavioral phase markers (Section 4.4). The broad and constrained sets differed both in material properties and in how positively they were experienced, and this design cannot separate the two; in a sensitivity model including the four rating differences, the interaction remained positive (β = +0.16, 95% CI [+0.003, +0.317], p = 0.046; N = 38) (Supplementary Material 5), so those differences do not account for it on their own, and we do not infer that either caused the association.

### 4.3 Implications for neuroergonomics

The findings extend wearable fNIRS research beyond task-evoked workload by showing that the association between a disposition and prefrontal hemodynamics can vary with occupational background, material context, and task progression during naturalistic design. Most applied fNIRS work in this tradition has targeted task-evoked workload, human-factors states, and neuroadaptive training (Hill and Bohil, 2016; Zahabi et al., 2020; Kia et al., 2021; Mark et al., 2022).

These findings suggest that future field applications of this type of physiological measure may need to model the person and the context jointly, rather than interpret the signal on the basis of a single individual or condition. Because the signal varied with the material set, the approach also lends itself to neuroergonomic evaluation of materials, tools, and workspace configurations rather than assessment of the worker in isolation. In practical terms, the material set functioned as a low-cost, manipulable environmental variable, suggesting a route from workload-oriented prefrontal fNIRS assessment (e.g., Mandrick et al., 2013; Causse et al., 2017; Midha et al., 2021) toward evaluating design environments themselves, for example by comparing candidate material palettes for a design workspace while modeling disposition and context jointly. Recent work has likewise extended fNIRS-based neuroergonomic evaluation to fast-paced applied team tasks (Zhang et al., 2026) and to team decision-making in engineering design (Yang et al., 2026), and a recent review advocates neurotechnology-informed, human-centered evaluation of designed environments (Fu et al., 2026). These implications are hypothesis-generating and require confirmatory work before operational use. The time-resolved analysis can also indicate when during an open-ended task such an association is most evident, which may motivate future studies of when support such as prompts, resources, or rest could be useful. Measuring design output was beyond the present scope; linking this process-level signal to design output remains for future work.

Methodologically, proportional-time normalization offers neuroergonomics a way to analyze self-paced, variable-duration naturalistic tasks whose trials cannot be aligned on an absolute clock. By mapping each trial from onset to completion onto a common process axis and combining this with time-resolved cluster-based inference, the analysis localized this association along an otherwise unsegmented process without imposing predefined stages. The same approach may be evaluated in other naturalistic, variable-duration activities studied in neuroergonomics, such as assembly, inspection, or open-ended creative work, where fixed-window averaging would obscure process-level structure.

### 4.4 Limitations and future confirmation

This study was not designed as a psychometric validation of the DAM. Rather, it provides preliminary evidence of an association between DAM Visualization scores and the prefrontal hemodynamic signal under specific material conditions. Because Visualization was assessed with a brief three-item self-report subscale of moderate reliability (α = 0.72), which likely attenuates the observed association through measurement error, the present findings should not be interpreted as physiological validation of the underlying construct. Replication in a larger sample with more comprehensive measurement of Visualization is needed. The two-site forehead montage (Fp1, Fp2) does not resolve specific brain regions and cannot fully separate cortical from scalp and systemic signals (Tachtsidis and Scholkmann, 2016), although short-separation regression substantially reduced the latter (Supplementary Material 2). In addition, the single 810-nm wavelength cannot separate oxy- and deoxyhemoglobin, so ΔHbT remains a relative hemodynamic index, and no inference is drawn about the underlying neural mechanism (Section 2.3). Because statistical adjustment for accelerometer-derived movement does not remove transient optical artifacts from the optical signal itself, residual motion contamination cannot be excluded. Color, piece count, and shape covaried across the material sets; from an ergonomics standpoint, material variety is thus a composite design variable combining visual variety (color), assembly freedom (piece count), and constructive possibilities (shape), and a factorial manipulation is needed to isolate the active component. This confound reflects a broader trade-off in naturalistic neuroergonomics between ecological validity and experimental control (Gramann et al., 2021). Finer hand and arm motion, such as pieces handled and reach frequency, was not recorded. Years of professional design experience were also not recorded; although occupation was adjusted for in all focal models and adjusting for age and sex left the results unchanged (Supplementary Material 5), these adjustments are not a substitute for directly measuring professional experience, which the planned confirmation study will record. In this sample, Visualization scores were concentrated toward the upper end (10 of 39 at the maximum), so the magnitude of the association is estimated less precisely, and a wider range of scores would sharpen it. Both the design-averaged estimate and the temporal cluster were preserved under zero-phase low-pass filtering, indicating robustness to the tested low-pass specifications (Supplementary Material 5). Trial durations ranged widely (≈124–1137 s), and proportional-time normalization stretches or compresses absolute-time hemodynamic dynamics differently across participants, so equal completion percentages need not correspond to comparable cognitive or physiological states (for example, under differential fatigue); the temporal cluster was nonetheless robust to duration trimming (Supplementary Material 5). The 60–79% window is an approximate range set by the cluster-forming threshold, so a confirmatory study can preregister an interval of about 60–80% and add behavioral phase markers to pinpoint it.

## 5 Conclusions

Consistent with prior work, Visualization was the only DAM dimension on which designers scored significantly higher than non-designers (d = 1.07). After adjustment for occupation, higher Visualization was associated with a larger broad–constrained Fp2 difference, driven by a positive association under the broad set and most evident at approximately 60–79% of the proportional timeline. The partial associations of occupation and Visualization with the broad–constrained Fp2 difference were in opposite directions, so adjusting for occupation increased the estimated positive association; this statistical suppression separates the visual-externalization disposition from the occupational label. The difference between Fp1 and Fp2 was not statistically significant, so the findings do not establish reliable right lateralization. The results require preregistered replication, but they suggest that wearable fNIRS combined with proportional-time analysis can help characterize how the association between Visualization and prefrontal hemodynamics varies with occupation, material context, and task progression during naturalistic design.

## Data Availability

The datasets presented in this study can be found in online repositories. The names of the repository/repositories and accession number(s) can be found below: OSF, https://doi.org/10.17605/OSF.IO/6VTD2 (Nagamori et al., 2026).
