# Supplementary material for: Material context moderates the occupation-adjusted association between Visualization and prefrontal hemodynamics during naturalistic design: an exploratory wearable fNIRS study
Source: Front Neuroergon. 2026 Sep 10;7:1928775. doi: 10.3389/fnrgo.2026.1928775 (PMC13601332; doi:10.3389/fnrgo.2026.1928775)
Supplement: Supplementary file 1 [file Data_Sheet_1.pdf]

Supplementary Material for: Material context moderates the occupation-adjusted association between Visualization and prefrontal hemodynamics during naturalistic design: An exploratory wearable fNIRS study

Supplementary Material

This supplementary material comprises, in order of first citation in the main text: the design attitude measure (DAM) items and their original subscale reliabilities (Supplementary Material 1); a signal-quality and interpolation summary (Supplementary Material 2); the between-channel comparison (Supplementary Material 3); the Freedman–Lane residual-permutation procedure and robustness checks for the focal interaction (Supplementary Material 4); further robustness and sensitivity analyses—the condition main-effect permutation procedure, demographic-adjustment and leave-one-out checks, and sensitivity analyses for motor/systemic and subjective-experience confounding, the unadjusted association, predictor distribution, task order, and frequency filtering (Supplementary Material 5); and the full design-averaged regression outputs and the time-resolved cluster-permutation results (Supplementary Material 6). The DAM items are reproduced in English from Ando et al. (2020, Table 2) for reader transparency; the present study administered the validated Japanese version (Yaegashi et al., 2022).

Focal model. Unless stated otherwise, analyses regress the within-participant broad − constrained difference in the design-averaged proportional signal on the focal predictors—grand-mean-centered Visualization and effect-coded occupation—by ordinary least squares ($N = 39$, $df = 36$; Section 2.5); 'occupation-adjusted' denotes inclusion of the occupation term.

Supplementary Material 1. Design attitude measure (DAM): English item content, scoring, and original reliability.

Subscale	Reliability	Code	Item
Experimentalism	α = 0.84	E1	When an idea fails I primarily focus on what I can learn from the experience.
		E2	Failed solutions provide a great opportunity for learning more about the problem.
		E3	I always focus on what I can learn from a failed idea.
		E4	When I receive feedback on a solution I stop to consider how the information should be used.
Optimism	α = 0.80	O1	Even toughest problems can be solved.
		O2	It is possible to create a solution for any problem.
		O3	I believe every problem can be solved.
Visualization	α = 0.77	V1	I prefer to use visuals to show how a solution will work.
		V2	I like to use visuals to explain my ideas to people.
		V3	Visuals are a great way to explain ideas to people.
Collaboration	α = 0.73	C1	I know that the best solutions come from sharing ideas.
		C2	I actively look for engagement with others when problem solving.
		C3	It is very important to reflect on ideas offered by others when finalizing a solution.
Empathy	α = 0.73	P1	I can easily see a problem through the eyes of those impacted by the

		situation.
	P2	When someone tells me how a problem affects them I can readily see the situation from their point of view.

Note. All items are rated on a six-point Likert scale (1 = strongly disagree, 6 = strongly agree), and each subscale score is calculated as the mean of its constituent items. Reliability coefficients are from the original validation study by Ando et al. (2020; $N = 2{,}348$). The present study administered the validated Japanese version reported by Yaegashi et al. (2022); internal consistency in the present sample is reported in Section 2.4.

Supplementary Material 2. Signal-quality and interpolation summary.

Condition	Median % (IQR)	Max %	Max consecutive (samples / s)	Retained / excluded
constrained	0.00 [0.00, 0.15]	4.88	14 / 1.4 s	39 / 0
broad	0.00 [0.00, 0.11]	12.14	21 / 2.1 s	39 / 0

Note. Interpolated samples are those flagged by the device software (noise rate ≠ 0) and linearly interpolated on the continuous signal (Section 2.3); the noise flag is shared between Fp1 and Fp2, so interpolation is identical for the two channels. Percentages are computed over each trial's analysis epoch across the 39 retained participants. All 39 participants contributed to both conditions, with no missing data at any proportional-time point; one participant was excluded for task noncompliance (Section 2.1). Within the 60–79% interval, interpolated samples occurred in only 6/39 (constrained) and 3/39 (broad) trials, making interpolation an unlikely explanation for the temporal cluster. Short-separation regression efficacy, computed from the raw 1-cm and 3-cm channels: the two signals were strongly correlated (median $r = 0.85$ at Fp2, 0.89 at Fp1), and the regression removed a median 74% (Fp2) and 80% (Fp1) of long-channel variance, including a median 62–64% of Mayer-wave (0.07–0.13 Hz) and 58–66% of cardiac-band (0.8–1.5 Hz) power, indicating substantial attenuation of superficial and systemic contributions (with some between-recording variation). Cortical activation is typically accompanied by increases in both oxy- and total hemoglobin (Kamran et al., 2016). HbT has also been used as a frontal hemodynamic measure in applied fNIRS research (Kim et al., 2022).

Distribution across participants. Flagged samples occurred at all in 17/39 constrained and 16/39 broad trials, exceeded 1% of samples in 3 and 2 trials, respectively, and exceeded 5% of samples in no constrained trial and one broad trial (12.14%; its raw and corrected traces are shown in Figure SM1); the per-participant proportion did not differ significantly between material sets (Wilcoxon signed-rank test, $p = 0.259$). Short-separation regression addresses superficial physiological contamination and is not a motion-correction method; only device-flagged samples were linearly interpolated (Section 2.3), so unflagged transient artifacts may remain, and statistical adjustment for accelerometer-derived head movement (Supplementary Material 5) does not remove such artifacts from the optical signal itself. The characterizations of HbT above serve as background only; we make no claim that the residual signal specifically reflects cortical activity.

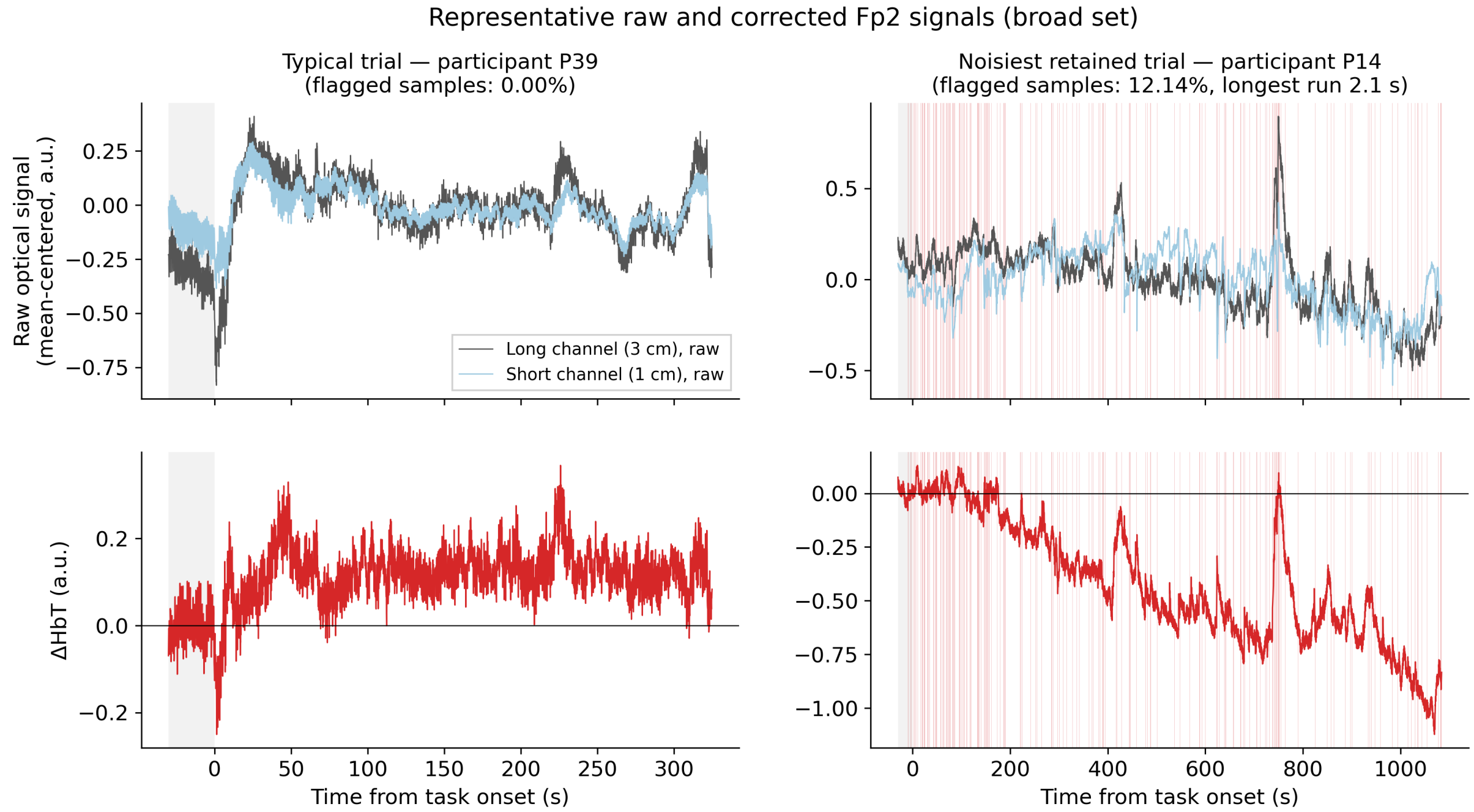

Figure SM1. Representative raw and corrected Fp2 signals (broad set). Top row: raw long-channel (3 cm) and short-channel (1 cm) optical signals (mean-centered for display). Bottom row: the ΔHbT signal carried forward to analysis (short-separation-adjusted, linearly detrended, baseline-corrected). Grey band: the 30-s eyes-closed rest used for baseline correction. Red bands: samples flagged by the device (noise rate ≠ 0) and linearly interpolated before analysis. Left: a typical trial (participant P39; 0.00% flagged). Right: the noisiest retained trial (participant P14; 12.14% flagged, longest consecutive run 2.1 s).

Supplementary Material 3. Between-channel comparison of the Condition × Visualization association.

**Between-channel test**	**Estimate / cluster**	**p**
Channel × Condition × Visualization (whole design)	β = +0.14 [−0.03, +0.32]	0.112
Channel difference (Fp2 − Fp1), temporal cluster (≈ 69–79%)	two-sided FL, n.s.	0.056

Note. Participant-level estimates from the design-averaged proportional signal (N = 39, ordinary least squares, df = 36; occupation-adjusted). The whole-design Channel × Condition × Visualization interaction tests whether the focal Condition × Visualization slope differs between channels. The time-resolved channel-difference test is a two-sided cluster-based permutation test (5,000 Freedman–Lane residual permutations preserving the Visualization–occupation structure). Under this test the Fp2 cluster was significant (60–79%, p = 0.013), whereas the channel difference (Fp2 − Fp1) did not reach significance (69–79%, p = 0.056).

Supplementary Material 4. Freedman–Lane residual permutation. At each proportional-time point the Condition × Visualization effect was the Visualization coefficient in the ordinary-least-squares regression of the within-participant broad − constrained difference on the focal predictors, converted to a t-statistic. The reduced model regressed the difference on occupation alone; its residuals were permuted across participants, added back to the fitted values, and the full model was re-estimated, preserving the Visualization–occupation structure. Within each permutation, the participant-level residual series were reordered by a single permutation applied identically across all 100 proportional-time points, so that each participant's residual time course was permuted as a unit and the within-participant temporal dependence was preserved. Clusters were formed where $|t| > 2.03$ (two-sided, df = 36) and summed to a cluster mass; the family-wise-error-

corrected p-value is the proportion of 5,000 permutations whose maximum absolute cluster mass equalled or exceeded the observed value.

Robustness of the focal interaction. Residuals of the design-averaged focal model were approximately normal (Shapiro–Wilk W = 0.97, p = 0.520), and homoscedasticity was not rejected (Breusch–Pagan p = 0.079). The interaction remained significant under the less conservative heteroscedasticity-consistent standard errors (HC0 p = 0.033, HC1 p = 0.040) but not under the more conservative ones (HC2 p = 0.055, HC3 p = 0.085); it also held under a 5,000-sample case-resampling bootstrap (95% CI [+0.02, +0.35]; Pr(β > 0) = 0.99).

Supplementary Material 5. Further robustness and sensitivity analyses. Condition main-effect permutation: the participant-level broad − constrained time courses were tested separately at Fp2 and Fp1 using two-sided one-sample sign-flip cluster permutations with 2,000 permutations.

Demographic-adjustment and influence (leave-one-out) robustness for Q1 and Q2.

Test	Specification	Estimate / p
Q1: Visualization (Designers − Non-designers)	unadjusted ANOVA	$F(1, 38) = 11.47$, $p = 0.002$ **
Q1 (as above)	+ age	$p = 0.004$ **
Q1 (as above)	+ sex	$p = 0.005$ **
Q1 (as above)	+ age + sex	$p = 0.016$ *
Q1 (as above)	+ age + sex, bootstrap Pr(Designers > Non-designers)	0.997
Q2: Condition × Visualization (Fp2)	occupation-adjusted base model	β = +0.188 [+0.036, +0.340], p = 0.017 *
Q2 (as above)	+ age	β = +0.191 [+0.039, +0.343], p = 0.015 *
Q2 (as above)	+ sex	β = +0.177 [+0.023, +0.330], p = 0.025 *
Q2 (as above)	+ age + sex	β = +0.181 [+0.026, +0.337], p = 0.024 *
Q2 (as above), leave-one-out	39 refits, sign	positive for all 39 (β = +0.10 to +0.22)
Q2 (as above), leave-one-out	39 refits, significance	raw $p < 0.05$ in 38/39

Note. Q1: one-way ANOVA / ANCOVA on the Visualization subscale ($N = 40$); bootstrap = 5,000 resamples of the age- and sex-adjusted estimated marginal-mean difference. Q2: design-averaged proportional-signal ordinary least squares (broad − constrained on the focal predictors; $N = 39$, df = 36), adding grand-mean-centered age and effect-coded sex as covariates. Leave-one-out omits each participant once and refits the occupation-adjusted focal Q2 model; the interaction sign was preserved in all 39 fits. Across these refits the interaction remained significant (p < 0.05) in 38/39 fits, reaching non-significance only when the single most influential participant was removed; by contrast, the effect within the fixed 60–79% interval remained significant in all 39 leave-one-out refits (all two-sided permutation p < 0.05), indicating that the effect within the fixed interval was robust to individual participants.

Duration-trimmed sensitivity analyses. Trial durations ranged from ≈124 to 1137 s. To test whether participants with extreme durations drive the focal results, the whole-design interaction and the fixed 60–79% window effect were re-estimated under three trimming rules. Visualization was re-centered within each subsample; models are occupation-adjusted as in the main analysis. Fixed-window p values are two-sided Freedman–Lane residual permutation p (20,000 permutations).

Trimming rule	N	Whole design: β [95% CI], p	Fixed 60–79% window: β [95% CI], permutation p
None (full sample)	39	+0.188 [+0.036, +0.340], p = 0.017	+0.292 [+0.126, +0.459], p = 0.0014
Any trial < 240 s excluded	27	+0.225 [+0.033, +0.417], p = 0.024	+0.342 [+0.131, +0.553], p = 0.0034
Mean duration within 10th–90th percentile ([230, 764] s)	31	+0.192 [+0.024, +0.361], p = 0.027	+0.317 [+0.127, +0.508], p = 0.0028
Any trial < 180 s or > 900 s excluded	31	+0.191 [+0.024, +0.357], p = 0.027	+0.311 [+0.123, +0.500], p = 0.0031

Note. The 240-s rule matches the internal data-quality marker used during acquisition; the percentile band and the 180/900-s rule are distribution-based alternatives, reported jointly to avoid dependence on any single cutoff. The fixed 60–79% interval is the sample-defined cluster held fixed (grid indices 60–79). The whole-design interaction was also robust to including trial duration as a covariate (see above).

Sensitivity analyses: alternative explanations, the unadjusted association, and predictor distribution.

Test	Specification	Result
Motor / systemic confound	Visualization × (B−C diff: accelerometer / gyroscope / pulse / duration), r	+0.17 / +0.28 / −0.21 / −0.03 (all p ≥ 0.08)
Motor / systemic confound	Q2 interaction + accelerometer + gyroscope + pulse covariates	β = +0.21, *p* = 0.013 *
Motor / systemic confound	Q2 interaction + trial-duration covariate	β = +0.19, *p* = 0.020 *
Subjective-experience confound	Visualization × (broad − constrained diff: difficulty / enjoyment / idea generation / material sufficiency), r	+0.01 / −0.10 / −0.16 / −0.24 (N = 38; all p ≥ 0.15)
Subjective-experience confound	Q2 interaction + (broad − constrained) differences in difficulty, enjoyment, idea generation, and material sufficiency	β = +0.16 [+0.003, +0.317], p = 0.046 * (N = 38)
Suppression / observability	Unadjusted (occupation-free) Q2 interaction	β = +0.11 [−0.03, +0.24], p = 0.116
Suppression / observability	Occupation-adjusted Q2 interaction	β = +0.19 [+0.04, +0.34], *p* = 0.017 *
Predictor distribution	Visualization: mean (SD), range	5.06 (0.79), 3.0–6.0
Predictor distribution	At ceiling (= 6.0)	10 / 39
Task-order robustness	Q2 interaction adjusted for task order (and Visualization × task order); 20 broader-first / 19 constrained-first	β = +0.20 [+0.04, +0.35], *p* = 0.014 *; task-order main effect *p* = 0.47; Visualization × task-order *p* = 0.25
Frequency filtering	None applied (short-separation regression and linear detrending only); focal test uses each participant's design average	Design-averaged estimate and the ≈60–79% cluster were preserved under zero-phase low-pass filtering (0.08–0.20 Hz; the effect within the same fixed window was significant in all 39 leave-one-out refits), indicating robustness to the tested low-pass specifications.

Note. The Q2 interaction is the Condition × Visualization effect on Fp2 (within-participant broad − constrained of the design-averaged proportional signal regressed on the focal predictors; *N* = 39). The subjective-experience confound model

uses N = 38: one participant was excluded because complete post-trial ratings across both conditions were unavailable; all recorded fNIRS trials were retained through the interpolation used in the main analysis. Covariates are within-participant (broad − constrained) differences in concurrently recorded accelerometer- and gyroscope-derived head movement, pulse rate, and trial duration; the unadjusted model omits occupation. The predictor distribution is from the $N = 39$ analysis sample.

Supplementary Material 6. Full results of the design-averaged regression models.

Model: predictor	β	SE	95% CI	*t* (df = 36)	p
Fp2 (broad − constrained): Intercept	+0.016	0.050	[−0.085, +0.118]	+0.33	0.745
Fp2 (broad − constrained): Visualization	+0.188	0.075	[+0.036, +0.340]	+2.52	0.017 *
Fp2 (broad − constrained): Occupation	−0.122	0.058	[−0.240, −0.003]	−2.09	0.044 *
Fp1 (broad − constrained): Intercept	+0.019	0.059	[−0.100, +0.138]	+0.33	0.743
Fp1 (broad − constrained): Visualization	+0.045	0.088	[−0.133, +0.222]	+0.51	0.614
Fp1 (broad − constrained): Occupation	−0.011	0.068	[−0.149, +0.128]	−0.16	0.876
broad (Fp2): Intercept	+0.107	0.041	[+0.024, +0.190]	+2.62	0.013 *
broad (Fp2): Visualization	+0.168	0.061	[+0.044, +0.292]	+2.75	0.009 **
broad (Fp2): Occupation	−0.128	0.048	[−0.225, −0.031]	−2.68	0.011 *
constrained (Fp2): Intercept	+0.091	0.035	[+0.021, +0.161]	+2.62	0.013 *
constrained (Fp2): Visualization	−0.020	0.052	[−0.125, +0.085]	−0.38	0.706
constrained (Fp2): Occupation	−0.006	0.040	[−0.088, +0.075]	−0.16	0.875

Note. Ordinary least squares on the design-averaged proportional signal (N = 39; df = 36), re-running the analysis in Section 2.5. broad − constrained denotes the within-participant difference; in the difference models the Visualization coefficient is the Condition × Visualization interaction and the Occupation coefficient is the Condition × Occupation interaction. The broad and constrained rows are the per-condition simple-slope regressions. Main-text values are rounded. In a model containing occupation but not Visualization, the Occupation × Condition interaction was not significant (p = 0.396); in the joint model, the occupation coefficient was negative and significant (p = 0.044), opposite in sign to Visualization, reflecting suppression given the Visualization–occupation correlation (r ≈ 0.52).

Time-resolved cluster-permutation results.

Analysis	Cluster (proportional time)	Cluster mass	max |t| (location)	FWE-corrected p
Fp2 (Freedman–Lane)	60–79% (positive)	63.2	4.11 (73%)	0.013 *
Fp2 (Visualization-label permutation)	60–79% (positive)	63.2	4.11 (73%)	0.014 *
Fp1 (Freedman–Lane)	none	—	—	No cluster survived
Condition main effect, Fp2 (sign-flip)	none	—	—	0.273 (n.s.)
Condition main effect, Fp1 (sign-flip)	none	—	—	1.000 (n.s.)

Note. Two-sided cluster-based permutation on the 0–100% proportional-time axis; cluster-forming threshold $|t| > 2.03$ ($df = 36$); cluster mass = summed t within a cluster; 5,000 permutations. Freedman–Lane = residual permutation preserving the Visualization–occupation structure; a simpler permutation of the Visualization labels gave a concordant cluster. The condition main-effect rows are one-sample sign-flip permutations (2,000) of the participant-mean broad − constrained time course ($df = 38$), independent of Visualization. No significant negative cluster was detected in any analysis.
